# How Reliable Is the Posterior Belly of the Digastric Muscle in Preventing Carotid Injury During Neck Dissection?

**DOI:** 10.7759/cureus.74231

**Published:** 2024-11-22

**Authors:** Harry May, Cynthia Schwartz, Yusuf Dundar

**Affiliations:** 1 Otolaryngology, Head and Neck Surgery, Texas Tech University Health Sciences Center School of Medicine, Lubbock, USA; 2 Otolaryngology, Head and Neck Surgery, Rutgers New Jersey Medical School, Newark, USA

**Keywords:** external carotid artery, neck dissection, posterior belly of the digastric muscle, surgical landmark, vascular injury

## Abstract

The posterior belly of the digastric muscle, referred to as the “resident’s friend," serves as a valuable anatomical landmark because identification of its location during head and neck surgery helps to secure vital structures.

A 53-year-old female was referred for an oral cavity mass with a biopsy confirmed squamous cell cancer. A physical exam revealed a 4 cm long and 2 cm wide right ulcerated oral tongue mass. Imaging findings demonstrated no evidence of pathological lymph nodes and distant metastasis. A surgical excision with elective neck dissection was planned along with submental island flap reconstruction. During the neck dissection, the external carotid artery (ECA) was noted to be passing over the posterior belly of the digastric muscle and then descending deeper than the stylohyoid muscle. No other vascular anomaly was identified. The surgery was completed without any complications, and the patient was discharged on post-operative day five.

It is necessary to be attentive to a potential course variation of the neurovascular structures underneath that could affect the credibility of the posterior belly of the digastric muscle as a trusted friend. This case report emphasizes the significance of identifying the rare variation where the posterior belly of the digastric muscle exposes the ECA to its lateral side.

## Introduction

Known as the "resident's friend," the posterior belly of the digastric muscle is a useful anatomical landmark during head and neck surgery because its identification helps secure vital structures [1]. The posterior belly of the digastric muscle overlays the internal and external carotid arteries, as well as the spinal accessory and hypoglossal nerves. Identifying the posterior belly of the digastric muscle early in neck surgeries helps the surgeon assess the depth and protect these vital structures, which are located deeper than the muscle. The only major vessel that lies superficial to the posterior belly of the digastric muscle is the common facial vein, which can be safely ligated without significant morbidity. The facial nerve is also located superior to the posterior belly of the digastric muscle. Because of these anatomical relationships, the posterior belly of the digastric muscle is called the “resident’s friend.”

However, the “resident’s friend” is not always a reliable guide, as there are many neurovascular variations in this surgical territory. Surgeons need to be meticulous and should never assume that the carotid vessel is always located beneath the digastric muscle. In this case report, we present a patient whose external carotid artery crosses laterally over the posterior belly of the digastric muscle.

## Case presentation

**Figure 1:**
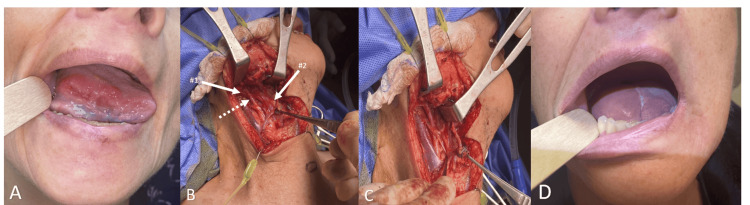
Case pictures A: an ulcerated lateral tongue mass; B: the right neck dissection where the ECA (dotted arrow) is located laterally, instead of medially, to the posterior belly of the digastric muscle proximal to mastoid process (white arrow #1) and its intermediate tendon (white arrow #2); C: the posterior belly of the digastric muscle was retracted superiorly to identify the internal carotid artery as well as courses of vessels at parapharyngeal space; D: patient’s recovery postoperatively for six months without visual complications. ECA: external carotid artery

The patient was a 53-year-old female who was referred for an oral cavity mass with a biopsy confirmed squamous cell cancer. Her social history showed no concerns regarding tobacco use or alcohol addiction except secondhand smoke exposure. She reported chronic irritation secondary to the denture. On physical exam, the right lateral ulcerated oral tongue mass was 4 cm long and 2 cm wide without palpable neck mass (Figure 1A). Neck CT with contrast did not show any evidence of pathologic lymph nodes, and positron emission tomography-computed tomography (PET-CT), and neck CT were negative for any obvious distant metastasis. With a clinical diagnosis of T3N0M0 oral cavity/oral tongue squamous cell cancer, we discussed her case at the multidisciplinary tumor board, and the recommendation was surgical excision with elective neck dissection as the standard of care. We discussed alternative reconstruction options, including healing by secondary intention, skin graft, submental island regional flap, supraclavicular island flap, radial forearm free flap, or anterolateral thigh free flap reconstruction. She is right-handed, works as a farmer, and does not want to have any morbidity in her extremities. She elected submental island flap reconstruction if intraoperative findings allow and anterolateral thigh-free flap as a backup option. A hemiglossectomy was performed, and a frozen section confirmed clear surgical margins. We assessed the hemiglossectomy defect, and she had a 6x4 cm oral tongue and floor of mouth defect. Then we planned a submental island accordingly and started neck dissection. ECA was noted to be passing over the posterior belly of the digastric muscle and then diving deeper than the stylohyoid muscle during the neck dissection (Figure 1B, 1C). Branches of the ECA, such as the superior thyroid, facial artery, and lingual artery, as well as the internal carotid artery (ICA), were identified to rule out any other vascular abnormalities. Also, parapharyngeal space was examined, and there were no other abnormalities or unusual variations identified. Surgery was completed without any complications and discharged on postoperative day five. 

The postoperative pathology report was consistent with moderately differentiated invasive squamous cell carcinoma with > 5 mm surgical margins. The tumor was measured 1.7 cm and 17 mm deep; lymphovascular invasion is present but perineural invasion is absent. A total of 28 lymph nodes were examined, and seven out of 28 had metastatic squamous cell cancer without any extranodal extension. Positive lymph nodes were at level 1B, 2A, and 2B stations, with the biggest lymph node measuring 14 mm at level 2A. We discussed her postoperative findings at the multidisciplinary head and neck tumor board and staged as pT3N2bM0 and recommended adjuvant chemoradiation based on pathologic stage and high-risk features. Post-treatment 12-month clinical examinations and scans are consistent with no evidence of disease. Post-treatment 12-month clinical exam showed appropriate articulation and excellent recovery (Figure 1D). 

## Discussion

The posterior belly of the digastric muscle serves as a crucial landmark in various procedures in head and neck surgery. In most cases, as ECA, its branches, and other vital neurovascular structures securely ascend medial to the posterior belly of the digastric muscle, this muscle is also known as the “resident’s friend.” However, this muscle can exhibit structural variations, including a split posterior belly of the digastric muscle or the absence of the muscle itself [1,2]. Moreover, it is also necessary to be attentive to a potential course variation of neurovascular structures, in our case the ECA, that could affect the credibility of the posterior belly of the digastric muscle as a trusted friend. There are a few reports of the ECA being laterally exposed instead of medially positioned to the ICA, but the ECA lateral to the posterior belly of the digastric muscle has been rarely discussed [3]. The anatomical variation we found is a noteworthy example to report; a research study investigating 1100 head sides of Japanese subjects found that only 0.37% of subjects had the ECA passing over the posterior digastric [4]. Head and neck surgery was the second-most implicated subspecialty of otolaryngology, and almost half of the medical cases related to otolaryngology were improper surgical performance, which entails a variety of intraoperative complications [5]. Still, a thorough examination of vital landmarks could mitigate confusion and minimize potential surgical risks. More importantly, it is crucial to proceed with caution and avoid having blind trust in the posterior belly of the digastric muscle.

## Conclusions

Vital structures in the neck, such as the internal jugular vein, ECA, spinal accessory nerve, and hypoglossal nerve, are underneath the digastric muscle's posterior belly. However, a rare course of the vital neurovascular structures uncovered by the posterior belly of the digastric muscle can trick surgeons and complicate neck dissection. This case report highlights the importance of identifying the rare variation where the posterior belly of the digastric muscle exposes the ECA to its lateral side, which could potentially pose serious surgical complications. To avoid unnecessary risks to patients, being mindful of rare cases and careful observation are essential.
